# Cell Death in the Kidney

**DOI:** 10.3390/ijms20143598

**Published:** 2019-07-23

**Authors:** Giovanna Priante, Lisa Gianesello, Monica Ceol, Dorella Del Prete, Franca Anglani

**Affiliations:** Kidney Histomorphology and Molecular Biology Laboratory, Clinical Nephrology, Department of Medicine - DIMED, University of Padua, via Giustiniani 2, 35128 Padova, Italy

**Keywords:** apoptosis, necrosis, regulated necrosis, kidney injury, tubular injury, glomerular injury

## Abstract

Apoptotic cell death is usually a response to the cell’s microenvironment. In the kidney, apoptosis contributes to parenchymal cell loss in the course of acute and chronic renal injury, but does not trigger an inflammatory response. What distinguishes necrosis from apoptosis is the rupture of the plasma membrane, so necrotic cell death is accompanied by the release of unprocessed intracellular content, including cellular organelles, which are highly immunogenic proteins. The relative contribution of apoptosis and necrosis to injury varies, depending on the severity of the insult. Regulated cell death may result from immunologically silent apoptosis or from immunogenic necrosis. Recent advances have enhanced the most revolutionary concept of regulated necrosis. Several modalities of regulated necrosis have been described, such as necroptosis, ferroptosis, pyroptosis, and mitochondrial permeability transition-dependent regulated necrosis. We review the different modalities of apoptosis, necrosis, and regulated necrosis in kidney injury, focusing particularly on evidence implicating cell death in ectopic renal calcification. We also review the evidence for the role of cell death in kidney injury, which may pave the way for new therapeutic opportunities.

## 1. Introduction

While naturally occurring cell death had already been observed many years ago, it was long considered a passive phenomenon and seen as an unavoidable endpoint of biological systems. Cells can remain stationary, supporting the relationships between an organ’s structure and function, or they can proliferate, sometimes becoming hypertrophic, or they can die. Regulation of the homeostatic balance between cell proliferation and cell death is important to the development and maintenance of multicellular organisms. 

The historical concept of programmed cell death has been associated with apoptosis because it is considered a form of suicide, based on a genetic mechanism. Any cell death other than apoptosis has generally been called “accidental cell death” [1]. Necrosis has consequently been described as accidental cell death [2] rather than as the result of definite pathways. The classic definition of necrosis is not really appropriate, because it does not always indicate a particular form of cell death. The term is often used to refer to changes secondary to cell death by any mechanism, including apoptosis. Many insults induce apoptosis at lower doses and necrosis at higher doses. Depending on the stimulus, apoptosis and necrosis could lie on a continuum of cell death, so the two forms are not mutually exclusive, and can coexist in many pathological conditions.

**Figure 1:**
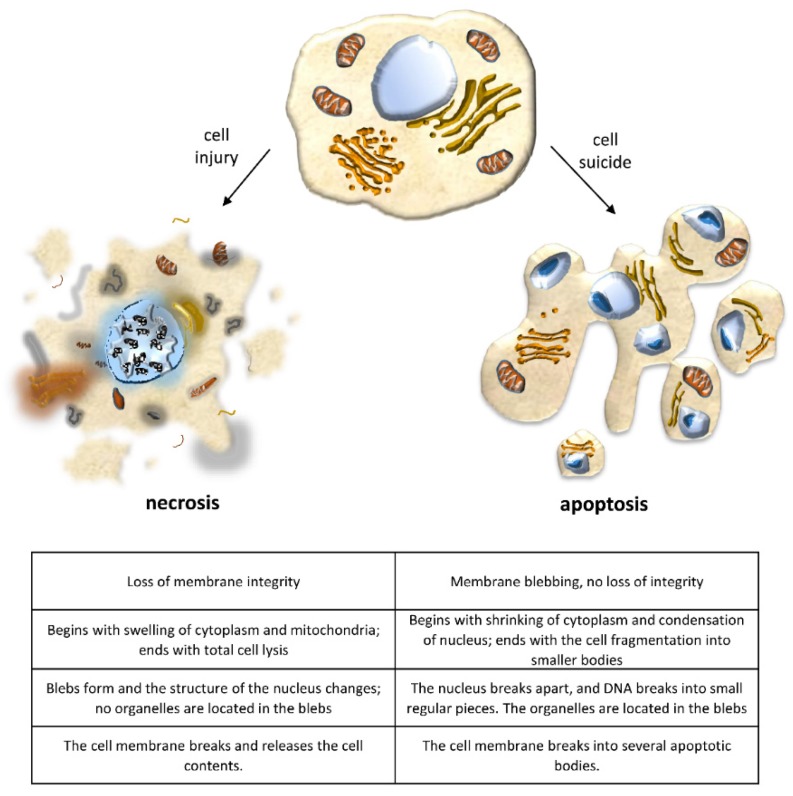
Changes in cell morphology that distinguish apoptosis from necrosis.

Cell death by apoptosis usually occurs in response to the cell’s microenvironment, and it is as fundamental to cellular and tissue physiology as cell division and differentiation. Attention to this form of cell death was prompted primarily by its crucial role in the normal embryonic development of higher vertebrates and in maintaining normal tissue homeostasis [3,4,5,6] by controlling cell numbers and eliminating nonfunctioning, damaged, or misplaced cells. As a result, and given that there are both pro- and anti-cell-death genes, the apoptotic pathway has been equated to programmed cell death (PCD). PCD is described as cell death occurring at a definite point in time during physiological development, based on an embedded genetic program that works like a clock. Because most examples of PCD happen by apoptosis, and apoptosis appears to be programmed by molecular events in the cell, the two terms are often used interchangeably. A now well-accepted concept of PCD includes clear examples that are not apoptosis, however [7,8,9]. Indeed, PCD can result in either a lytic or a nonlytic morphology, depending on the signaling pathway, whereas apoptosis is a nonlytic and typically immunologically silent form of cell death. Programmed lytic cell death is highly inflammatory, and necrosis is distinguished from apoptosis because of the related inflammatory response due to the rupture of the plasma membrane and release of intracellular content, including cellular organelles and highly immunogenic proteins (Figure 1).

It has now been established that necrosis is not an accidental, passive, unregulated form of cell death, but like apoptosis, it can be governed by a “regulated” mechanism, meaning a death with the classic morphological features of necrosis but genetically determined [5,10,11].

At a molecular level, the best-characterized pathway of regulated necrosis (RN) is necroptosis, a receptor-interacting protein kinase (RIPK)-based necrotic cell death [9,12,13,14,15]. Several other specialized forms of regulated necrosis have been described, however, such as ferroptosis [16], pyroptosis [17], parthanatos [18,19], mitochondrial permeability transition-dependent regulated necrosis (MPT-RN) [20], pyronecrosis [21,22], and NETosis, a process based on the rapid release of so-called neutrophil extracellular traps (NETs) [23]. All of these cell death pathways occur independently of an RIPK, or they can occur in the presence of RIPK inhibitors, often highlighting overlapping functions and pathways. Thus, cell death, be it by apoptosis or necrosis, is now considered to be regulated cell death (RCD) rather than a PCD. RCD implies an active participation of the cell in its own death through the activation of a genetically encoded death program, which specifies the means for starting a process that leads to the point of no return. Autophagy is a newly described, highly regulated cell death typified by the markers of a specific pathway [24], and it is as necessary as apoptosis for keeping the kidney healthy.

It is very important to be aware that every cell is “programmed” to die in response to an appropriate stimulus. Disruption of the signaling pathways or aberrant triggering of the processes that regulate physiological cell death due to extracellular causes, infections, toxins and toxicants, gene mutations, etc., may lead to abnormal cell functioning, which can become manifest in a wide array of human diseases. Gaining further insight into cell death mechanisms and a better understanding of the molecular processes involved will lead to a better characterization of a disease’s etiology and pathogenesis. Cell death pathways can also be manipulated, targeting their clinical management as a way to develop new treatment approaches.

## 2. Kidney Injury and Cell Death

Although the term apoptosis was first introduced in the early 1970s by Kerr et al. [25], Glucksmann et al. had already described the morphological alterations associated with the process in kidney cells in the early 1950s [26].

It is now well accepted that apoptosis is an integral part of normal kidney functioning. As in other tissues, there is no inflammatory response in apoptotic cells, and their smaller fragments (apoptotic bodies) in the kidney providing these bodies are promptly ingested by neighboring cells and are degraded in lysosomes or eliminated via the tubular lumen. In fact, various types of cells may be involved in this tissue maintenance process, including epithelial cells. Phagocytes recognize and engulf apoptotic cells before their membrane is damaged, protecting surrounding tissues and cells from the damaging effects of the release of intracellular contents. If apoptotic cells are not ingested by phagocytes or epithelia, however, the cells proceed to a necrotic phase (called secondary necrosis), and their contents can spill into the extracellular space, causing inflammation and leading to inflammation-mediated kidney injury. Attempts to interfere with apoptosis (by certain caspase inhibitors, for instance) may trigger necrosis and consequent inflammation-mediated kidney injury [27].

The rate of apoptosis in the kidney is particularly intense in the developmental age [28,29,30,31]. Given the complexity of the renal microenvironment, cell death in the kidney is decoded in terms of the organ as a whole, not separately by its tubular, glomerular, interstitial, or endothelial compartments. Like all complex organisms, moreover, the kidney needs a physiological cell death modality: cell proliferation and cell death are strictly linked to keep the overall number of cells constant, eliminating cells that are damaged or no longer necessary at each stage of development, based on endogenous or exogenous factors. Developing kidneys are known not only to contain apoptotic cells [28,32], but also to express high levels of several apoptosis-related genes [33,34,35,36,37,38,39].

The mature mammalian kidney is a quiescent organ with little or no mitotic activity, and little or no apoptosis has been found in adult human kidneys. Thus, the nephrogenesis process (the formation of new nephrons) is limited to the period of embryonic development in humans. However, the mature kidney is capable of cellular proliferation, and in certain circumstances renal cells (like differentiated neurons) can divide. Several observations now point to the existence of adult kidney stem cells being implicated in both homeostatic tissue maintenance and functional recovery after injury [40,41,42,43,44,45,46,47,48].

In the case of injury in the adult kidney, cell death may occur in different compartments (the tubular and glomerular) and different types of cells, including the proximal and distal tubular cells and endothelial and glomerular cells [27]. Renal cell death is central to the pathophysiology of renal diseases. Renal cell loss is rarely a consequence of apoptosis, but rather of regulated necrosis, or simply the flushing of detached living cells.

Several common renal insults have been shown to disrupt kidney autophagy, including ischemia, toxic injury, and inflammation. Dysregulated, excessive, or defective autophagy is implicated in numerous disease states. Dysregulated autophagy leads to chronic inflammation and autoimmune diseases. Excessive autophagy can contribute to the expansion of malignant cells in renal cell cancer. Insufficient autophagy is a result of renal ischemia and facilitates cell death [49]. The death rate of renal cells might be abnormally high in nephropathies, promoting cell loss, as in acute tubular necrosis (ATN), acute rejection, necrotizing glomerulonephritis, or renal atrophy. Conversely, the cell death rate may drop (with an abnormal accumulation of cells) in cases of proliferative glomerulonephritis, polycystic renal disease, and neoplasia, for instance [50,51,52,53].

### 2.1. Tubular Cell Injury

Cell death in renal disease has been investigated primarily through the mechanism of tubular damages. In acute renal failure, cell death may be a direct consequence of exposure to harmful stimuli. Many renal insults, such as toxic injury or ischemia, mainly affect tubular epithelial cells and the metabolically very active proximal tubular segment in particular. Tubules are responsible for the reabsorption and secretion of several solutes, and injury to this nephron segment is the main mediator of acute kidney injury (AKI), which determines a rapid decline in renal function.

Apoptosis increases in the event of an acute unilateral ureteral obstruction (UUO) due to a physical obstruction or congenital anomalies. This causes renal growth impairment and tubular atrophy, primarily in the distal tubular epithelium, but also in the proximal renal tubules, resulting in hydronephrosis and renal failure [54,55,56,57,58,59]. Stretching, ischemia, and oxidative stress following ureteral obstruction are primary causes of tubular cell apoptosis. Increased apoptosis also activates cell infiltration, interstitial cell proliferation, and interstitial fibrosis [59,60,61]. Intriguingly, mild injury triggers apoptosis, and tubulointerstitial atrophy after UUO results from cell deletion by apoptosis. This leads to phagocytosis of the apoptotic bodies by neighboring tubular cells and direct apoptotic cell shedding into the tubular lumen, reestablishing homeostasis. When injury is severe, however, necrosis is more likely to be the dominant model of cell loss [61,62,63,64].

Renal ischemia followed by reperfusion (I/R) initiates apoptosis in the proximal tubular cells [27,65,66,67]. I/R injury is known to be caused by ischemia, and then recovery of the blood flow unexpectedly worsens the damage. Renal tubular epithelial cell apoptosis is the key pathophysiological alteration occurring in I/R, and it defines the extent of the damage to kidney function. I/R injury is related to several inflammatory reactions, among which endothelial cell activation, the expression of adhesion molecules, the adhesion, aggregation and activation of leukocytes and platelets, the production of oxygen free radicals, and cellular calcium increase, as well as with the apoptosis mediated by these processes. AKI caused by I/R is a clinical syndrome that prompts kidney dysfunction and leads to a high mortality rate [68,69].

The consecutively hypoxic and oxidative stress evoked by renal I/R has been shown to enhance autophagy in several rodent models. The role of autophagy after renal I/R injury is still debated, however, and both protective and detrimental properties have been proposed [70].

Apoptosis seems to be common in post-transplant acute and chronic renal failure due to I/R injury [71,72,73,74,75,76,77]. In acute rejection, apoptosis occurs in the renal tubular epithelium, leading to tubular atrophy [78]. Chronic renal allograft rejection develops gradually, suggesting persistent low-grade injury, with a sustained and irreversible loss of renal function accompanied by clinical signs of proteinuria and hypertension [79,80].

During chronic kidney disease (CKD), the depletion of tubular cells by apoptosis gradually increases, contributing to the tubular atrophy and renal fibrosis associated with the progression of CKD [56,81,82,83,84,85]. Necrosis occurs in CKD as well, and the relative involvement of the two death mechanisms in cell loss depends on the balance of regulatory events. The dynamics of cell death during the early and intermediate stages of CKD have remained unclear, however. In an animal model that mimicked the progression of CKD in humans (rats undergoing subtotal nephrectomy), the authors demonstrated that both necrosis and apoptosis caused tubular injury. Since the RIPK3-regulated pathway was predominant with respect to the caspase-3 regulated pathway, the authors concluded that necrosis was the primary mechanism mediating renal tubular epithelial cell loss in the early and intermediate stages of chronic renal damage [86].

Tubular atrophy and tubular epithelial cell apoptosis have a role in diabetic kidney disease, although vascular and glomerular injuries are considered the main features of this condition [87,88,89]. Hyperglycemia triggers the generation of free radicals and oxidative stress in the tubular cells, and reactive oxygen species (ROS) are well-known important mediators of several biological responses, including proliferation, extracellular matrix deposition, and apoptosis [90].

Evidence of apoptosis has also been found in toxic renal exposure. The large luminal membrane surface area of proximal tubular cells makes them particularly susceptible to toxicants. Both toxicants and natural toxins are associated with altered renal apoptosis and affect several cellular factors. Studies with arsenic trioxide showed that low quantities of this agent prompted Bax/Bak-dependent apoptosis, while higher doses triggered MPT and apoptotic/necrotic cell death [91]. Thes studies showed that different cell death modalities may coexist within the same injury and that interference with specific signaling pathways or critical cell functions might result in cell killing by a distinct process. Various heavy metals induce apoptosis in renal cells via diverse mechanisms and molecular pathways [92,93,94,95,96,97,98]. For example, free cadmium accumulates in mitochondria, blocking the respiratory chain and culminating in mitochondrial dysfunction and the release of free radicals, which triggers caspase cascade and apoptosis. Antineoplastic agents are among the drugs that can trigger renal epithelial cell apoptosis. Cisplatin induces apoptosis in already low concentrations, resulting in cell loss, and this effect appears to be mediated by the generation of ROS. Oxidative damage to mitochondrial lipids and proteins increases with caspase-3 activity [27,99,100,101]. Moreover, the disruption of intracellular Ca^2+^ homeostasis or the induction of mild oxidative stress might mediate the apoptosis-inducing effects of these chemicals. In rat renal proximal tubules, cytochalasin D and dithiothreitol also caused apoptosis with associated cytoskeletal disorganization [102,103]. Antibiotics may have the potential to induce apoptosis, too. Gentamicin was found to induce apoptosis in renal distal tubules in the acute phase of injury and in the proximal tubules during the recovery phase [104,105]. Natural toxins from contaminated food and water supplies also pose a potential risk of renal apoptosis [106].

### 2.2. Glomerular Cell Injury

Cell death has also been documented in the diseased glomerulus [107,108,109,110,111]. Harrison et al. [107] were the first to report finding apoptotic bodies in human glomerulonephritis based on light and electron microscopy of kidney biopsies.

Apoptosis in glomerulonephritis appears to reduce hypercellularity during the repair process, controlling the size of the glomerular population and clearing excess cells. Apoptosis is required for the recovery of normal glomerular function [109,110,112,113]. In an experimental model of glomerulonephritis, however, the number of apoptotic glomerular cells was found to increase with the progression of glomerulosclerosis [114]. Apoptotic cell accumulation in the glomeruli has also been found to correlate with the glomerular sclerosis index and apoptotic index (the number of apoptotic cells divided by the number of normal cells) and with a decline in kidney function [114,115]. These results suggest that apoptosis is one of the mechanisms of glomerular cell depletion during progressive glomerulosclerosis.

In proliferative glomerulonephritis, on the other hand, the lack of a compensatory increase in cell death gives rise to an accumulation of cells, with glomerular hypercellularity due to mesangial and endocapillary cell proliferation [108,116,117,118,119]. During the chronic proliferative stage of systemic lupus erythematosus (SLE), apoptotic cells’ number declines, while the number of proliferating cells increases, resulting in an imbalance in tissue homeostasis. An impaired removal of apoptotic bodies also indirectly contributes to the pathogenesis of SLE. Histone-bound DNA complexes, which have high affinity for the glomerular basement membrane, are carried out from apoptotic cells and accumulated in the glomerulus, triggering an immune response and causing glomerular damage [120,121,122,123,124,125,126,127,128,129].

In crescentic glomerulonephritis (CGN), disease progression is related to fibrosis of the glomerular crescents and renal interstitium. A number of different cell types, such as epithelial cells, fibroblasts, monocytes and macrophages, have been involved in the development of glomerular crescents and their progression to fibrosis. Proliferating macrophages as well as proliferating parietal epithelial cells seem to be the master contributors to this type of lesion [130,131,132], however, indicating that the glomerular proliferative index is more important than apoptosis alone in CGN.

Idiopathic nephrotic syndrome is the result of podocyte impairment. Basement membrane denuding and podocyte detachment and loss have been implicated in several human nephrotic syndromes, including focal and segmental glomerulosclerosis, minimal change disease, glomerulonephritis, and diabetic nephropathy [133,134,135,136]. Remarkably, not only resident glomerular cells but also infiltrating leukocytes might be eliminated by apoptosis in the glomeruli. Indeed, apoptotic bodies have been found to be particularly prominent in glomeruli containing numerous neutrophils, proving that apoptosis is a homeostatic mechanism that enables hypercellular glomeruli to return to normal [108,109,110,137,138].

### 2.3. Necrosis/Regulated Necrosis and the Kidney

Renal cortical necrosis is the death of tissue in the outer portion of the kidney (cortex) resulting from the blockage of the small arteries supplying blood to the cortex, and it causes AKI. The cause is usually a significantly diminished renal arterial perfusion secondary to vascular spasm, microvascular injury, or intravascular coagulation. Renal cortical necrosis is generally extensive, though focal and localized forms do occur. In most cases, the medulla, the juxtamedullary cortex, and a thin rim of subcapsular cortex are spared [139].

Renal papillary necrosis is a disorder in which all or part of the renal papillae die. It is characterized by coagulative necrosis of the renal medullary pyramids and papillae brought on by several associated conditions and toxins synergistically promoting the onset of ischemia. Renal papillary necrosis can lead to secondary infection of desquamated necrotic foci, stone formation, and/or the separation and eventual sloughing of papillae, resulting in acute urinary tract obstruction. The clinical course of renal papillary necrosis depends on the degree of vascular impairment, the presence of associated causal factors, the patient’s general health, any bilateral involvement, and specifically, the number of papillae affected [140].

The biochemical signaling pathways that trigger necrosis have been investigated in detail in recent years. It is now clear that RN is a genetically driven process that strongly contributes to the pathophysiology of kidney injury.

Cell death by RN involves RIPK pathway-mediated rupture of the plasma membrane caused by a complement-related membrane attack complex, exotoxins, or cytotoxic T cells. Necrotic cell death is thus accompanied by the release of immunogenic cellular components collectively known as damage-associated molecular patterns (DAMPs) [141,142], which cause severe tissue damage, leading to systemic inflammation and organ injury or failure. Immune cell necrosis (i.e., NETosis or pyroptosis) is another component of necrotic renal lesions. This means that any causal factors triggering the RN signal pathways and the release of inflammatory mediators could be mutually enhancing and self-amplifying, leading to further renal cell loss, kidney atrophy, and scarring. The extremely proinflammatory effect of necrosis is very important in the kidney transplantation setting and in AKI, when inflammation occurs mostly together with renal cell necrosis (necroinflammation), as in necrotizing glomerulonephritis, thrombotic microangiopathy, and ATN [143,144]. RN modalities such as necroptosis, ferroptosis, parthanatos, and MPT-RN may be mechanistically distinct, but their damage to tubular segments and multicellular functional units may be synchronized, because otherwise they would only kill single cells in the tubular compartment [67,145,146,147,148]. Interestingly, the localization of tubular injury may differ in the several forms of renal damage. For example, tubular injury is variable in ischemic lesions, acting on short pieces of the proximal straight tubule and focal areas of the ascending limb of Henle’s loop. In toxic forms, tubular damage is more continuous along all segments of the proximal tubule.

In the early phases of I/R injury, reduced oxygen supply to metabolically active tubular epithelial cells lowers oxidative metabolism and depletes cell supplies of high-energy phosphate compounds. Reperfusion restores the oxygen supply and improves oxygen radical formation, resulting in mitochondrial impairment. Neutrophil infiltration participates in this process via NET formation and further histone release into the extracellular space due to tubular cell necrosis [143,149]. The innate immune response arises soon after I/R injury and involves neutrophils, natural killer cells, and macrophages. Together with the tubular epithelial cells, macrophages produce proinflammatory cytokines, thus contributing to injury. The histones released kill more tubular cells through direct cytotoxic effects, possibly interfering with normal mitochondrial function or altering lysosome function, thus resulting in cell membrane alteration and disorganized protein synthesis. The mechanism of histone cytotoxicity is probably due to the polycationic nature of histones and their capacity to bind to the anionic moiety cell walls [149]. Moreover, histones that are immunologically inert when they are within the nucleus exert DAMP effects once released into the extracellular space [143]. Concomitant with the injury, there is shedding of viable and necrotic cells into the tubular lumen, and as the lesion progresses, cell proliferation could also intensify in an effort to replace neighboring cells and repair tubular cell injury [150].

In immune complex diseases such as crescent glomerulonephritis, renal vasculitis, or antiglomerular basement membrane disease, cell necrosis triggers massive glomerular inflammation with cytokine and chemokine expression, together with the release of dangerous intracellular molecules. The influx of neutrophils accelerates this process, with a subsequent inflammatory response (NETosis) in the capillaries of the glomerular tuft. This loop triggers a massive parietal epithelial cell hyperplasia, followed by basement membrane rupture and plasma leakage from disrupted glomerular capillaries and then crescent formation [151,152,153,154].

In either tubular or glomerular injury, a mild form of the same insult can lead to apoptosis, while a severe form can lead to necrosis. The pathway followed by the cell therefore depends on both the nature and the severity of the insult, sometimes evolving from an apoptotic to a necrotic form of cell death.

### 2.4. Cell Death and Crystal Nephropathies

The kidney is susceptible to crystal formation, as mineral secretion and urine concentration favor supersaturation, which can give rise to several acute and chronic kidney disorders related to crystal deposition or formation inside the kidney, referred to as crystal or crystalline nephropathies and renal stone disease [155,156,157,158,159,160].

Tubular crystallopathies result from precipitates inside the tubular lumen. The dynamics of crystal deposition determine the outcomes of kidney injury, i.e., AKI or CKD. A sudden onset of crystal formation causes cell necrosis and inflammation leading to AKI, whereas a chronic dynamic of crystal formation causes plugs in distal tubules or collecting ducts, leading to persistent tubule obstruction and hence CKD.

Apart from the urine concentration of both minerals and regulators of crystallization, the different types of crystal-induced renal disease are determined not only by the physicochemical properties of the crystals but also by the type of signaling pathway triggered by the crystals. Once crystals have formed in the tubular lumen, they contribute to kidney injury mainly through a direct or indirect cytotoxic effect, the underlying molecular mechanisms of which are largely unknown. Furthermore, crystals elicit inflammation and inflammation-driven cell necrosis in an auto-amplifying loop that is referred to as necroinflammation [143].

Calcium oxalate, calcium phosphate, and other crystals of various composition are known to be capable of inducing cell death, especially in renal proximal tubule cells [155]. Their effect might depend on their size. Nanosized crystals primarily cause apoptotic cell death, whereas micron-sized crystals cause necrotic cell death. Nanosized crystals may be internalized and transferred into lysosomes, thus causing damage that can trigger apoptosis. Alternatively, crystals can pass through pores into the nucleus, prompting DNA cleavage into regular fragments, an important characteristic of apoptotic cell death.

Micron-sized crystals on cells may cause irregular injury of the cell membrane and local strong physical stress, resulting in necrotic cell death. Released inflammatory factors by necrotic cells lead to cell membrane rupture that in turn causes an imbalance in cell osmotic pressure and consequently the sudden massive destruction of lysosomes accompanied by hydrolytic enzyme release [157].

Various crystals can also enter cells via a process of phagocytosis [159,161]. Phagosomes fuse with lysosomes in an attempt to digest the crystals. However, either amorphous calcium released by lysosomes into the cytosol or indigestible lysosome particles trigger necroptosis.

Crystals deposited in the kidneys act as intrarenal DAMPs, promoting intrarenal inflammation and contributing to further tubular injury and subsequent renal dysfunction [100,162]. These molecules can activate Toll-like receptors, which results in inflammasome activation in immune renal cells. Tubular cell necroptosis or ferroptosis due to calcium oxalate internalization can contribute to promoting inflammation [147,148]. Crystal-induced DAMPs also include histones that in large amounts are released into extracellular space by necrotic cells. Due to their strong basic charge, histones can potentially disrupt plasma membranes of neighboring intact cells, a process that increases the number of dying tubular cells and thus aggravates kidney injury [149,160].

The importance of cell death in pathological soft tissue calcification is well documented [163]. Such calcifications usually consist of calcium phosphate salts (including hydroxyapatite), but they sometimes contain calcium oxalates, too, as in calcium nephrolithiasis. In the kidney, the presence of necrotic tubular cells has been associated with renal cortical calcification, a rare condition usually due to severe cortex destruction and any condition causing acute and prolonged shock [164]. The role of cell death in the more common medullary nephrocalcinosis - microscopic renal crystal deposition in the tubular lumen (intratubular nephrocalcinosis) or interstitium (interstitial nephrocalcinosis) -, frequently associated with nephrolithiasis remains unclear. In two in vitro models of nephrocalcinosis obtained by exposing wild-type or Glial cell-derived neurotrophic factor (GDNF)-silenced human renal tubular cells to an osteogenic medium, it was recently shown that apoptosis and necroptosis respectively triggered renal cell calcification even before calcium phosphate crystal deposition [165,166] and mimicked vascular cell calcification [167,168,169,170]. The authors speculated that if cell death is an important event in the pathogenesis of renal ectopic calcification, any damage that shifts the balance between cell survival and cell death toward the latter could (in conjunction with a particular renal milieu) give rise to interstitial nephrocalcinosis and ultimately to kidney stones [171].

## 3. Targeting Renal Cell Death

In the undeniably complex picture of the mechanisms involved in cell death, under certain conditions, markers of apoptosis and necrosis may be found simultaneously, meaning that more than one cell death mechanism can be activated at the same time [146,172,173,174]. While there may be signs of different cell death pathways being involved, one pathway is usually the fastest and most effective. It is important to understand the interplay between different cell death pathways, especially with a view toward targeting these pathways for therapeutic purposes.

Elucidating the precise mechanisms behind cell death is essential to the development of new drugs. Numerous cellular factors have been proposed to regulate cell death response following a variety of induction mechanisms in numerous cell types, but the role of many of these factors depends both on the signal triggering a given cell death process and on the type of cell in which the response is induced.

Apoptosis and RN are characterized by distinct morphological, cell biological, and biochemical features. These two forms of cell death can occur at the same time in the same kidney compartment. They are not mutually exclusive and coexist in many renal pathological conditions [27,67,175]. The occurrence of either may depend on the intensity of the triggering events. For instance, renal ischemia may kill cells by either apoptosis or necrosis. The proportion of cells killed by each mechanism may also vary from one individual to another, because a part of the cell population predetermined to die by apoptosis might be rescued by interference with the genetic program, or apoptotic cells and their debris might be rapidly removed by phagocytes. Alternatively, apoptosis may cause secondary necrosis, as in prolonged kidney injury, in which case the plasma membrane of apoptotic cells may break down, thus acquiring a necrotic morphology.

**Figure 2:**
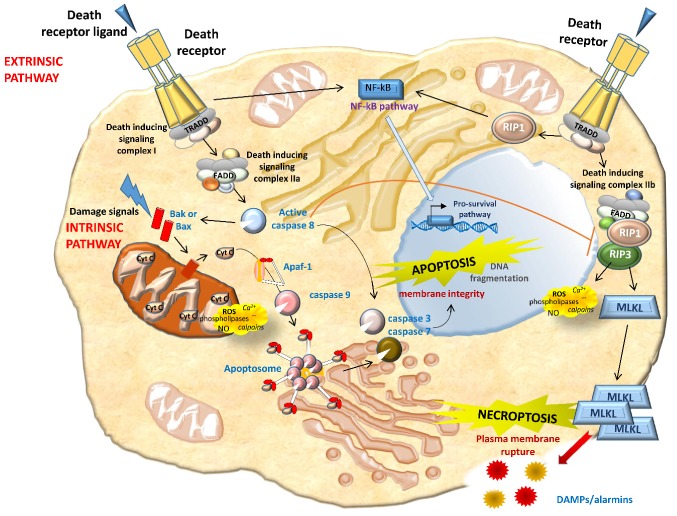
Overview of key molecular pathways of apoptosis and necroptosis. Apoptosis can start via intrinsic pathways (perturbation of intracellular homeostasis) or extrinsic pathways (death receptor binding). In the former case, cell stress leads directly (or via mediators, such as Bax and Bak) to mitochondrial outer membrane permeabilization, resulting in the release of apoptogenic factors, including Cytochrome *c*, which binds Apaf-1 to stimulate caspase-9 via apoptosomes. Bcl-2-related proteins induce apoptosis (e.g., Bax) or protect against it (e.g., Bcl-2). As for the extrinsic pathway, death receptor binding guides the recruitment of adapter proteins such as TRADD (TNFR-associated death domain), forming complex I. While complex I promotes cell survival via NF-кB activation, its transition to a secondary cytosolic complex, complex II, mediates cell death. Complex II is formed through the association of complex I with FADD (Fas-associated death domain). The formation of complex IIa promotes the activation of apoptosis in a caspase-8-dependent manner. Upon inhibition of caspase 8, complex IIb promotes necroptosis. Caspase-8 or caspase-9 activation subsequently triggers executioner caspases, such as caspase-3, -6, and -7. The cleavage of receptor-interacting protein kinase (RIPK) 1 and 3 by caspase-8 leads to apoptosis, whereas their phosphorylation triggers necroptosis in conditions of caspase-8 inhibition. RIPK1 and RIPK3 activation in turn causes the recruitment of the executioner mixed-lineage kinase domain-like protein (MLKL), which is phosphorylated by RIPK3 and initiates structural changes, leading to its insertion into the plasma membrane and channel formation. MLK channels increase Na^+^ influx, osmotic pressure, and membrane rupture, ending in cell death. Membrane rupture promotes the release of intracellular contents and endogenous damage-associated molecular patterns (DAMPs) and/or preformed proinflammatory molecules (alarmins). Through RIPK1 kinase activity, a wide range of necrotic mediators are activated in the execution phase of necrotic cell death, including reactive oxygen species (ROS), calcium (Ca^2+^), calpains, cathepsins, phospholipases, and ceramide.

Apoptosis occurs in three different phases: Initiation, effector, and final. The initiation phase is dependent upon stimuli, and two pathways can be identified, either extrinsic or intrinsic. In the extrinsic (death receptor-mediated) pathway, the ligation of death receptors determines the enrolment and activation of caspase-8. Caspase-8 further activates downstream caspases leading to apoptosis. Caspase-8 also triggers the intrinsic pathway to intensify the apoptotic cascade and inhibits necroptosis (Figure 2). In the intrinsic (mitochondrial) pathway, pro-apoptotic Bcl-2 family proteins Bax and Bak create pores on the mitochondrial outer membrane, determining the release of apoptogenic factors, such as Cytochrome *c* (Cyt C). In the cytosol, Cyt C binds to and stimulates conformational modifications in the adaptor protein Apaf-1, thus leading to the enrolment and activation of caspase-9. Caspase-9 further activates executioner caspases to elicit apoptosis. Notably, other components of the Bcl-2 protein family, such as Bcl-xL and Bcl-2, prevents pore formation in healthy cells by binding to Bax and Bak. Initiating factors include Tumor necrosis factor (TNF) receptors and ligands, growth factors, and changes in the extracellular matrix. Oxidative stress plays an important role in renal apoptosis. Either by acting as signal transduction molecules or by directly causing cellular damage, ROS activate apoptosis at multiple steps in the cell death pathway and lead to the damage of cellular macromolecules, including DNA, proteins, and lipids [27,91] (Figure 2).

**Figure 3:**
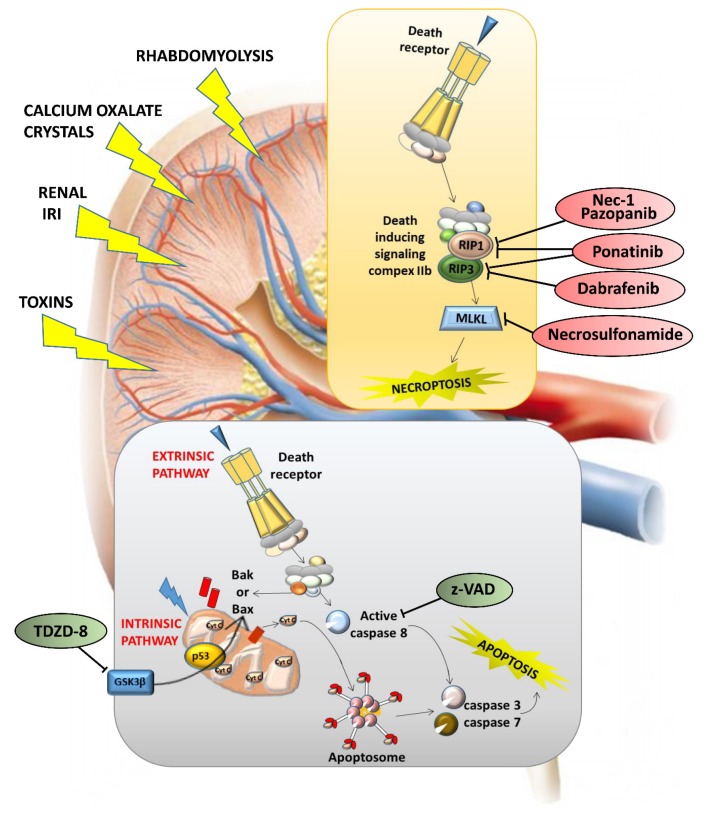
Targeting apoptosis and necroptosis in kidney lesions. Different kidney lesions activate different cell death modalities: z-VAD (z-Val-Ala-Asp fluoromethyl ketone) has been found to protect renal function, and the effect of the pan-caspase inhibitor z-VAD on experimental renal ischemia-reperfusion (I/R) injury was to reduce serum urea levels, thereby preventing inflammation. TDZD-8 (4-benzyl-2-methyl-1,2,4-thiadiazolidine-3,5-dione) has been found to inhibit ischemia-induced activation of GSK3β (glycogen synthase kinase 3 beta), Bax, and caspase 3, thus ameliorating tubular and epithelial cell damage and significantly protecting renal function.

In the final phase, which is common in extrinsic and intrinsic pathways, apoptotic cells show cytoplasmic shrinkage, chromatin condensation (pyknosis), nuclear fragmentation (karyorrhexis), and plasma membrane blebbing, culminating with the formation of apoptotic bodies. Since initiators and effectors of apoptosis are often unique to a particular cell or induction mode and take action upstream from the final common phase, cells committed to an apoptotic pathway may be rescued by specific therapeutic interventions. Several therapies targeting the apoptotic pathway have shown beneficial effects in many in vitro and in vivo models. Caspase inhibitors, such as z-VAD (z-Val-Ala-Asp fluoromethyl ketone), reduced apoptosis and improved organ function in several AKI models [176,177]. TDZD-8 (4-benzyl-2-methyl-1,2,4-thiadiazolidine-3,5-dione), a pharmacological inhibitor of the powerful proapoptotic kinase GSK3β (glycogen synthase kinase 3 beta), reduces proximal tubular epithelial cell apoptosis and has positive influences on the kidney by inhibiting inflammation and increasing renal cell proliferation [178,179]: This makes it a rational option in human trials designed to prevent or treat AKI (Figure 3).

RN may interact with apoptosis at various molecular and cellular levels, but both forms of cell death involve pathological changes in the mitochondria. In renal and other cells, the mitochondria are crucial sites for integrating intrinsic and extrinsic apoptotic signals. Bax, a known classic pro-apoptotic protein taking effect via mitochondrial membrane permeabilization, has been shown to regulate MPT-RN as well, again by affecting mitochondrial dynamics [180]. It is important to bear in mind, however, that mitochondria are not involved in some RN pathways [20]. Necroptosis is generally considered a mitochondrion-independent form of RN. Very recent studies, however, have shown that in TNF-induced necroptosis, ROS induction was RIPK3-dependent and well correlated with necroptosis. Using mitochondrial respiration inhibitors and mitochondrial depletion, the authors showed that a TNF-induced increment of aerobic respiration accounted for ROS induction in necroptosis [181]. As they are the point where cell injury and death converge, the mitochondria may be promising targets for therapy.

Similarly to extrinsic apoptosis, necroptosis begins with the activation of death receptors such as Fas and TNF-α receptor 1 (Figure 2). Provided that caspase-8 (a key inhibitor of necrosis) is inactive, RIPK1 activates RIPK3, which in turn activates the mixed-lineage kinase domain-like protein (MLKL). MLKL oligomerizes and translocates to the plasma membrane, determining membrane rupture. RIPK1 is thought to be essential to necrosis induced by the Fas ligand and TNF- α. Necrostatin-1 (Nec-1) is an RIPK1 inhibitor that prevents the death of TNF-α-treated Fas-associated death domain (FADD)-deficient cells [182,183] (Figure 3). In addition to Nec-1, a number of necroptosis inhibitors have been reported [184,185,186,187,188,189,190,191,192], and some of them have been approved by the Food and drug administration (FDA), such as dabrafenib (a selective RIPK3 inhibitor), pazopanib (for RIPK1), and ponatinib (for both RIPK1 and RIPK3) [189,190] (Figure 3). In light of the multiple initiating pathways upstream, manipulating downstream necroptosis mediators such as MLKL may be more effective [182] (Figure 3). Although necroptosis inhibitors have been used in clinical trials, there are several issues to consider. These drugs may be useful, given their current clinical use as anticancer agents, but whether their toxicity-related side effects are admissible for patients with necroptosis-associated renal diseases remains to be seen. Many necroptosis inhibitors have yet to be extensively explored, so the efficacy and safety of these potential drugs should be further validated. Necroptosis also prompts the release of unprocessed intracellular contents, such as DAMPs or various preformed proinflammatory molecules (alarmins) stored inside the cell [142,193,194,195]. These proinflammatory molecules activate innate immunity, prompting the production of more proinflammatory cytokines and consequently more necrosis in renal tissue. If not opposed at an early stage, this self-amplifying process can lead to systemic inflammation and remote organ injury or even organ failure. In theory, the RN pathways can therefore be targeted therapeutically to block these inflammatory agents, and this should suffice to slow and even cancel RN [16,182,196,197,198]. It is essential to consider a combination of therapies capable of blocking multiple regulated cell death pathways, either simultaneously or at different time points, to ensure cell survival and renal function.

Necroptosis and apoptosis undoubtedly coexist in the pathophysiological process of AKI. Despite the limitations of separating the two cell death pathways, therapeutic interventions that primarily inhibit apoptosis have the potential to minimize renal dysfunction and accelerate recovery after AKI. The effectiveness of anti-apoptosis therapies has confirmed the contribution of apoptosis to AKI, which should not be neglected. Preclinical studies have identified several pathways resulting in RN that could be modulated successfully in AKI in vivo by drugs or interventions targeting the molecular pathways. In practice, specific therapies need to intercept events occurring upstream from cell death, so early intervention is essential. Unfortunately, this strategy might not be feasible in a large proportion of cases, because AKI is usually asymptomatic in the early stage.

## 4. Conclusions

Despite the difficulties of classifying cell death modalities in categorized patterns, great efforts have been made in recent years to do so in kidney injury. The description of new regulated cell death modalities, the realization that they may coexist in the same organ, and the discovery of inhibitors of the various types of cell death have raised hopes for therapeutic interventions in diseases characterized by massive cell death, such as AKI. Unfortunately, there have been no changes in clinical practice to date because it is difficult to translate these results into clinical trials in the absence of convincing preclinical evidence.
